# DSM-5 posttraumatic stress symptom dimensions and health-related quality of life among Chinese earthquake survivors

**DOI:** 10.1080/20008198.2018.1468710

**Published:** 2018-05-03

**Authors:** Gen Li, Li Wang, Chengqi Cao, Ruojiao Fang, Ping Liu, Shu Luo, Jianxin Zhang, Brain J. Hall, Jon D. Elhai

**Affiliations:** a Laboratory for Traumatic Stress Studies, CAS Key Laboratory of Mental Health, Institute of Psychology, Chinese Academy of Sciences, Beijing, China; b Department of Psychology, University of Chinese Academy of Sciences, Beijing, China; c College of Psychology and Sociology, Shenzhen University, Shenzhen, China; d Department of Psychosomatic Medicine, People’s Hospital of Deyang City, Deyang, China; e Global and Community Mental Health Research Group, Department of Psychology, Faculty of Social Sciences, University of Macau, Taipa, Macau (SAR) China; f Department of Health, Behavior and Society, Johns Hopkins Bloomberg School of Public Health, Baltimore, MD, USA; g Department of Psychology, University of Toledo, Toledo, OH, USA; h Department of Psychiatry, University of Toledo, Toledo, OH, USA

**Keywords:** PTSD symptom dimensions, health-related quality of life, DSM-5; China, earthquake, dimensiones de síntomas de TEPT, calidad de vida relacionada con la salud, DSM-5; China, terremoto, PTSD症状维度, DSM-5; 中國, 健康相关的生活质量, 地震, • Our study provides knowledge on how different symptom dimensions of DSM-5 PTSD are associated with physical and psychosocial HRQoL.• PTSD dysphoric arousal symptoms were found to significantly relate to physical HRQoL.

## Abstract

It has been well-documented that posttraumatic stress symptoms cause impairments in health-related quality of life (HRQoL). Until now we have little data on how DSM-5 PTSD symptom dimensions relate to different aspects of HRQoL. Clarifying this question would be informative to improve the quality of life of PTSD patients. This study aimed to investigate the effects of dimensions of a well-supported seven-factor model of DSM-5 PTSD symptoms on physical and psychosocial HRQoL. A total of 1063 adult survivors of the 2008 Wenchuan earthquake took part in this study nine years after the disaster. PTSD symptoms were measured by the PTSD Checklist for DSM-5 (PCL-5). HRQoL was measured by the Medical Outcomes Survey Short Form-36 (SF-36). The associations between PTSD symptom dimensions and HRQoL were examined using structural equation models. Dysphoric arousal symptoms were found to significantly relate to physical HRQoL. Other symptom dimensions were not associated with HRQoL. Our findings contribute to the relationship between DSM-5 PTSD and HRQoL, and carry implications for further clinical practice and research on trauma-exposed individuals.

## Abstract (es)

La evidencia previa indica que los síntomas de estrés postraumático causan déficits en la calidad de vida relacionada con la salud (CVRS). Hasta ahora, tenemos pocos datos sobre cómo las dimensiones de los síntomas del TEPT del DSM-5 se relacionan con diferentes aspectos de la CVRS. Aclarar esta pregunta ayudaría a mejorar clínicamente la calidad de vida de los pacientes con TEPT. Este estudio tuvo como objetivo investigar los efectos de las dimensiones de un modelo bien respaldado de siete factores de los síntomas del TEPT según el DSM-5 sobre la CVRS física y psicosocial. En este estudio participaron mil sesenta y tres supervivientes adultos del terremoto de Wenchuan en 2008, nueve años después del desastre. Los síntomas del TEPT se midieron mediante el Lista de Evaluación del TEPT para el DSM-5 (PCL-5), y la CVRS se midió mediante el Cuestionario de Salud en formato abreviado-36 (SF-36). Las asociaciones entre las dimensiones de los síntomas de TEPT y la CVRS se examinaron utilizando modelos de ecuaciones estructurales. Los síntomas de alerta disfórica se relacionaron significativamente con la CVRS física, mientras que las otras dimensiones de síntomas no se relacionaron con la CVRS.

Conclusiones: Nuestros hallazgos aportan conocimiento sobre las relaciones entre el TEPT según el DSM-5 y CVRS, y tienen implicaciones para la práctica clínica y la investigación en individuos expuestos al trauma.

## Abstract (zh)

已有文献证明，创伤后应激症状会导致与健康相关的生活质量（HRQoL）受损。 到目前为止，我们几乎没有关于DSM-5 PTSD症状维度如何与HRQoL的不同方面相关的数据。 澄清这个问题将有助于临床上提高PTSD患者的生活质量。 本研究旨在考察得到良好支持的DSM-5 PTSD症状的七因子模型维度对 躯 体和社会心理HRQoL的影响。 2008年汶川地震灾害发生9年后，有一千六百三十名成人幸存者参加了这项研究。 通过DSM-5的PTSD检查表（PCL-5）测量PTSD症状，并且通过《医疗结果调查简表-36（SF-36）》来测量HRQoL。 使用结构方程模型检查PTSD症状维度与HRQoL之间的关联。 发现痛苦觉醒症状与身体HRQoL显著相关，而其精神痛苦性唤起维度与躯体HRQoL无关。 我们的研究结果有助于了解DSM-5创伤后应激障碍与HRQoL之间的关系，并对创伤暴露个体的进一步临床实践和研究带来启示。

## 1. Introduction

Posttraumatic stress disorder (PTSD) is a mental disorder precipitated by exposure to a traumatic event. According to a recent WHO survey, the lifetime prevalence of PTSD was 3.9% in the general population and 5.6% among individuals who reported trauma history (Koenen et al., 2017). PTSD is associated with significant psychological distress, functional disability, and decreased quality of life (Schnurr, Lunney, Bovin, & Marx, 2009). A meta-analytical study reported a large effect association between PTSD and multiple domains of quality of life (Olatunji, Cisler, & Tolin, 2007), including health-related quality of life (HRQoL).

HRQoL is a multi-dimensional concept consisting of physiological, psychological, and functional aspects of well-being (Senneseth, Alsaker, & Natvig, 2012). According to several researchers (e.g. Gladis, Gosch, Dishuk, & Crits-Christoph, 1999), HRQoL reflects a functioning component of quality of life and directly affects subjective well-being. Clarifying the relationship between PTSD symptoms and HRQoL would provide valuable suggestions for improving quality of life among individuals experiencing traumatic events. People with PTSD symptoms usually face physical and emotional problems, which may further impair normal functioning and HRQoL (Schnurr et al., 2009). For example, emotional numbing symptoms of PTSD could lead to difficulties in expressing emotions, and in turn reduce psychosocial HRQoL (Samper, Taft, King, & King, 2004). Symptoms like nightmares or sleep disturbance cause physical health problems and undermine physical HRQoL. Schnurr and Green (2004) have proposed an integrative model to explain the relationship between PTSD and health outcomes like HRQoL. According to this model, PTSD may affect HRQoL through a complex process including psychological, biological, and attentional mechanisms. For example, PTSD symptoms may increase health risk behaviours like smoking or substance abuse, and consequently affect HRQoL. Empirical studies have found that poorer HRQoL was associated with PTSD symptoms or PTSD diagnosis across a range of traumatic events (e.g. Aversa et al., 2013; Pupo, Serafim, & De Mello, 2015; Senneseth et al., 2012; Wang, Cao, Wang, Zhang, & Li, 2012). As PTSD is a complex clinical syndrome with high heterogeneity (Zoellner, Pruitt, Farach, & Jun, 2014), different predominant symptom dimensions of PTSD may have unique effects on different aspects of HRQoL (Giacco, Matanov, & Priebe, 2013; Monson, Caron, Mccloskey, & Brunet, 2017). Several studies found that hyperarousal symptoms of PTSD rather than other symptom dimensions were most related to impairments in quality of life (Giacco et al., 2013; Pupo et al., 2015). Another study found an association with emotional numbing symptoms (Taylor, Wald, & Asmundson, 2006). A longitudinal study found PTSD symptoms were more strongly correlated with psychosocial than physical HRQoL (Schnurr, Hayes, Lunney, McFall, & Uddo, 2006). Additional work on HRQoL also supports unique effects of different PTSD dimensions (Maguen, Stalnaker, McCaslin, & Litz, 2009): only PTSD dysphoric arousal factor was related to physical HRQoL, and only PTSD avoidance and emotional numbing factors were related to psychosocial HRQoL (Wang et al., 2012). These studies focusing on the relationship between PTSD symptoms and HRQoL would help further our understanding on the heterogeneity of PTSD symptom presentations and psychopathology of PTSD. For example, revealing the relationship between different PTSD symptom dimensions and HRQoL could provide support for the heterogeneity of PTSD symptom presentations, which means PTSD symptoms play different roles in functioning impairments and should not be treated as homogeneous. Additionally, these studies could provide support to external validity for current PTSD symptom structure models and improve our knowledge on the psychopathology of PTSD. Moreover, these findings will benefit the development of more effective interventions focusing on specific symptoms to improve HRQoL.

Most current studies on the relationships between PTSD and HRQoL were based on the fourth edition of the *Diagnostic and Statistical Manual of Mental Disorders* (DSM-IV; American Psychiatric Association, 1994). In the recently released DSM-5 (American Psychiatric Association, 2013), three new PTSD symptoms were added (distorted blame, pervasive negative emotional state, recklessness) and several symptoms were revised (e.g. trauma-related amnesia, negative beliefs). Symptoms were categorized into four clusters: re-experiencing, avoidance, negative alterations in cognitions and mood, alterations in arousal and reactivity. Many studies have been conducted to determine the symptom structure of DSM-5 PTSD (Armour, Műllerová, & Elhai, 2016), among which a seven-factor hybrid model consisting of intrusion symptoms, avoidance, negative affect, anhedonia, externalizing behaviours, anxious arousal, and dysphoric arousal (Armour et al., 2015) was found to best capture dimensions of DSM-5 PTSD symptoms across adults (most recently in Mordeno, Go, & Yangson-Serondo, 2017) and youth (most recently in Wang et al., 2017) exposed to various trauma types. The intrusion factor contains all current Criterion B symptoms (intrusive thoughts, nightmares, flashbacks, emotional cue reactivity, physiological cue reactivity), and avoidance factor contains all current Criterion C symptoms (avoidance of thoughts and reminders). The negative affect factor is composed of trauma related amnesia (D1), negative beliefs (D2), distorted blame (D3), and pervasive negative emotional state (D4). The anhedonia factor is composed of lack of interest (D5), feeling detached (D6), and inability to experience positive emotions (D7). The externalizing behaviour factor consists of irritable or aggressive behaviour (E1) and reckless or self-destructive behaviour (E2). The anxious arousal factor consists of hypervigilance (E3) and exaggerated startle (E4), while the dysphoric arousal factor consists of difficulty concentrating (E5) and sleep disturbance (E6). As indicated by Pietrzak et al. (2015), the seven-factor model may provide greater specificity in understanding associations between DSM-5 PTSD and quality of life. There are no current studies that evaluate how the seven dimensions of DSM-5 PTSD symptoms related to different aspects of HRQoL.

Our current study aimed to accumulate knowledge on the relationships between DSM-5 PTSD and HRQoL to further our understanding of psychopathology and clinical treatments of PTSD. We investigated the association between DSM-5 PTSD symptom dimensions and HRQoL in a predominantly adult sample which experienced the 2008 Wenchuan earthquake in Sichuan Province, China. Our hypothesis was that different symptom dimensions of PTSD are differently associated with psychosocial and physical HRQoL.

## 2. Methods

### 2.1. Participants and procedures

In 2008, a catastrophic earthquake measuring 8.0 on the Richter scale hit Wenchuan in Sichuan Province, China. Hanwang Town was one of the areas that suffered the most severe damage and more than 5000 people were killed in the earthquake. Our sample was recruited from five rebuilt communities located in this town. We conducted our survey in July 2017, more than nine years after the earthquake. Our sampling procedures were as follows: (1) One member in each household was randomly selected as participants; (2) All participants were between 16 and 65 years old during the survey, and all personally experienced the 2008 earthquake; (3) Those with major psychosis (e.g. schizophrenia and organic mental disorders) were excluded. The investigation was conducted by trained clinical psychologists, psychology undergraduates, and graduate students. Before the participants completed self-reported questionnaires, the aims of our study were explained in detail by the investigators. We obtained written informed consent from all participants. The study protocol was approved by the Institutional Review Board of the Institute of Psychology, Chinese Academy of Sciences.

A total of 1074 people were recruited into our study. Two of them were excluded because of missing data on all PTSD symptoms, and nine of them were excluded because of more than 20% missing data on HRQoL. The final analytic sample was 1063: 341 (32.1%) males and 700 (65.9%) females. Age ranged from 16 to 65 years (Mean = 51.1, *SD* = 10.0). Regarding ethnicity, nearly all were Han (98.6%) and 1.4% were other ethnicities. With respect to educational level, 722 (68.0%) did not complete high school, 326 (30.6%) completed high school (including equivalency). In terms of marital status, 904 (85.0%) were married, 146 (13.7%) were unmarried (single/divorced/separated/widowed). Trauma exposure features of the sample are summarized in Table 1.

**Table 1.** Trauma exposure of the sample.

Trauma exposure	*n*	Percentage (%)
Trapped under rubble (yes)	127	11.9
Injured (yes)	149	14.0
Disabled due to injuries (yes)	42	4.0
Participated in rescue efforts (yes)	450	42.3
Witnessed a death of someone (yes)	711	66.9
Exposed to mutilated bodies (yes)	416	39.1
Lost at least one family member (yes)	299	28.1
Family member injured (yes)	397	37.3
Lost a friend or neighbour (yes)	836	78.6
Lost livelihood due to the earthquake (yes)	383	36.0

*N* = 1063.

### 2.2. Measures

Earthquake-related trauma exposure was measured by a 10-item questionnaire, and each item was rated with either 0 (no) or 1 (yes). Details of the questionnaire are shown in Table 1. DSM-5 PTSD symptoms were assessed with the PTSD Checklist for DSM-5 (PCL-5; Blevins, Weathers, Davis, Witte, & Domino, 2015). The PCL-5 is a self-report checklist of 20 PTSD symptoms defined in the DSM-5. Each item is rated on a five-point Likert scale reflecting severity of a particular symptom from 0 (not at all) to 4 (extremely) during the past month. The PCL-5 scale has demonstrated good reliability and validity (Blevins et al., 2015). The Chinese version of PCL-5 was adapted by translation and back translation, and has been previously used in traumatized Chinese samples (Wang et al., 2017). In this current sample, Cronbach’s α was 0.95 for the total scale.

Health related quality of life was measured by the Medical Outcomes Survey Short Form-36 (SF-36; Ware & Sherbourne, 1992). The SF-36 is a 36-item self-report checklist which consists of eight subdomains of HRQoL. Four domains reflect physical HRQoL (physical functioning, role-physical, bodily pain, general health); four domains reflect psychosocial HRQoL (role-emotional, social functioning, mental health, fatigue). Scores of each subdomain were standardized from 0 to 100. The SF-36 scale showed good validity and reliability (McHorney, Ware, & Raczek, 1993). The Chinese version of SF-36 has been widely used in Chinese samples (Li, Wang, & Shen, 2003). The mental health and fatigue subscales of the SF-36 demonstrate spurious relationships with the PCL (Schnurr et al., 2006). Therefore, we used only role-emotional and social functioning subscales as indicators of psychosocial HRQoL; higher scores indicate better functioning.

### 2.3. Statistical analysis

We performed data analysis using Mplus 7.0. In our sample, 86 (8.1%) were missing one PCL-5 item, 25 (2.4%) were missing two or three PCL-5 items, 201 (18.9%) were missing one SF-36 item, and 90 (8.5%) were missing two to six SF-36 items. Missing data on the PCL-5 and SF-36 were estimated using maximum likelihood (ML) procedures (Schafer & Graham, 2002). Two structural equation models (SEM) were constructed to examine associations between PTSD symptom dimensions and physical/psychosocial HRQoL  (see Supplementary Figures 1 and 2). The data were not normally distributed. Thus, we performed mean and variance-adjusted weighted least squares estimation (WLSMV) to adjust for non-normality in the SEM models (Muthén & Muthén, 2007). In the first model, physical HRQoL measured as a latent factor by physical functioning, role-physical, bodily pain, and general health subscale scores was regressed on seven PTSD symptom clusters. Regression coefficients were calculated to reflect the associations between each symptom cluster and HRQoL. In the other model, psychosocial HRQoL measured as a latent factor based on role-emotional and social functioning subscale scores was set as the dependent variable instead. The SEM analyses followed a two-step approach (Vasterling, 2008). First, measurement models were computed to test the adequacy of latent variables in explaining the corresponding observed items. Second, structural models were specified to examine the association between PTSD symptom dimensions and HRQoL. According to Hu and Bentler (1999), an acceptable fit is evidenced by RMSEA ≤ .08, SRMR ≤ .08, CFI ≥ .90, and TLI ≥ .90, and an excellent fit is evidenced by RMSEA ≤ .05, SRMR ≤ .08, CFI ≥ .95, and TLI ≥ .95. PTSD symptom dimensions were simultaneously included as independent variables. Demographic variables such as age, sex, marital status, and education level were treated as covariates in all analyses based on their potential associations with HRQoL (Fang et al., 2015).

## 3. Results

The mean score on the PCL-5 was 19.0 (*SD* = 15.4), ranging from 0 to 80. Using the DSM-5 diagnostic algorithm of at least one re-experiencing, one avoidance, two negative alterations in cognitions and mood, and two alterations in arousal and reactivity symptoms of at least moderate (2 or higher) severity, 169 (15.9%) participants were identified as probable PTSD cases. Mean scores, standard deviations, and Pearson correlations for 26 observed variables (i.e. 20 items of the PCL and six subscales of the SF-36) are detailed in Supplementary Table 1.

According to results of CFA, measurement models of the physical HRQoL model (χ^2^ (326, *N =* 1063) = 1909.45, CFI = .954, TLI = .947, RMSEA = .069) and the psychosocial HRQoL model (χ^2^ (275, *N =* 1063) = 1679.35, CFI = .958, TLI = .950, RMSEA = .071) all demonstrated acceptable fit to the data, which means all latent factors were well-measured by corresponding items. Both SEM demonstrated good fit to the data, indicated by χ^2^ (316, *N =* 1063) = 856.30, CFI = .983, TLI = .980, RMSEA = .041 (90% CI: .038–.044) for the physical HRQoL model, and by χ^2^ (265, *N =* 1063) = 707.50, CFI = .985, TLI = .982, RMSEA = .040 (90% CI: .037–.044) for the psychosocial HRQoL model. Table 2 shows the results for SEM. Only dysphoric arousal was significantly associated with physical HRQoL (β = −0.52, *p* = .008), while the other PTSD symptom dimensions were not significantly related to physical or psychosocial HRQoL. The significant result remained significant after Bonferroni correction for multiple comparisons (α = 0.05/2 = 0.025).

**Table 2.** Summary of structural equation analyses examining relation between the seven PTSD factors and psychosocial and physical health-related quality of life.

Dependent variable	Predictor	*r*	B	SE(B)	β
Physical HRQoL	In	−0.43**	0.05	0.24	0.07
	Av	−0.40**	0.03	0.06	0.04
	NA	−0.47**	−0.26	0.41	−0.33
	An	−0.47**	0.07	0.20	0.11
	EB	−0.50**	0.31	0.98	0.43
	AA	−0.52**	−0.20	0.70	−0.30
	DA	−0.58**	−0.33	0.13	−0.52**
Psychosocial HRQoL	In	−0.46**	−0.34	0.67	−0.26
	Av	−0.44**	0.02	0.15	0.02
	NA	−0.57**	−0.41	1.12	0.31
	An	−0.58**	−0.14	0.51	0.13
	EB	−0.63**	−1.69	2.76	−1.38
	AA	−0.57**	1.12	1.97	0.98
	DA	−0.60**	−0.42	0.30	−0.39

*N* = 1063. In = Intrusion; Av = Avoidance; NA = Negative Affect; An = Anhedonia; EB = Externalizing Behaviours; AA = Anxious Arousal; DA = Dysphoric Arousal. Sex, age, educational level, and marital status controlled as covariates.

*p < .05.

**p < .01.

## 4. Discussion

We investigated the relationships between seven symptom dimension of a newly refined factor model of DSM-5 PTSD symptoms and two aspects of HRQoL in an epidemiological sample of Chinese earthquake survivors nine years after a destructive earthquake. People with greater PTSD dysphoric arousal symptoms reported poorer physical HRQoL. We found age and education level were significantly associated with both physical and psychosocial HRQoL. Older people have low physical HRQoL (β = −0.251, *p* < .001) and psychosocial HRQoL (β = −0.175, *p* = .002). Education can be a protective factor for physical HRQoL (β = 0.19, *p* < .001) and psychosocial HRQoL (β = 0.19, *p* < .001). Our results were partly consistent with previous findings based on DSM-IV PTSD symptoms. A longitudinal study also found PTSD arousal symptoms were predictors of lower HRQoL four years later (Pupo et al., 2015). Prior work also found that dysphoric arousal symptoms significantly predict impairments in physical HRQoL (Wang et al., 2012). Our findings were consistent with a recent DSM-5 based study finding correlations between dysphoric arousal symptoms and impairments in physical functioning (Pietrzak et al., 2015). Our results suggested that dysphoric arousal could be the core symptom which affects quality of life in a Chinese population with PTSD symptoms. This is consistent with the literature that suggests somatization is common among Chinese samples (Hall et al., in press). In the current study, we provided new knowledge for understanding the relationship between PTSD and HRQoL by revealing the associations between different PTSD symptom dimensions with HRQoL. In our current study, we did not find any symptom dimension of PTSD was associated with psychosocial HRQoL. This result is contrary to many previous studies which find PTSD symptoms are significantly associated with reduced psychosocial HRQoL (e.g. Taylor et al., 2006). One possible explanation of this result may be the measurement of psychosocial HRQoL. The SF-36 scale was most often used to measure HRQoL. In previous studies, psychosocial HRQoL was measured by role-emotional, social functioning, mental health, and fatigue subscales of SF-36. However, as suggested previously (Schnurr et al., 2006), there are spurious relationships between PCL and the mental health and fatigue subscales of the SF-36. Some items of the two subscales are very similar to PCL items. For example, the mental health subscales contain items like ‘felt so down in the dumps that nothing could cheer you up’ or ‘felt downhearted and blue’. In PCL-5, there are very similar items: ‘persistent negative emotional state (D4)’ and ‘lack of interest (D5)’. Therefore, we excluded these two subscales from measurement of psychosocial HRQoL, and the association between PTSD and psychosocial HRQoL was no longer strong enough to reach significant level.

Our study indicated that dysphoric arousal symptoms developed after trauma exposure, consisting of difficulty concentrating and sleep disturbance, are associated with physical functioning problems and impaired physical quality of life. Some researchers proposed that dysphoric arousal symptoms are associated with general distress which is shared by mental disorders other than PTSD. Dysphoric arousal symptoms are not unique to PTSD, but are included in disorders like generalized anxiety disorder or major depression (Elhai et al., 2011). As suggested by some researchers (Spitzer, First, & Wakefield, 2007), dysphoric arousal should be excluded from PTSD diagnostic criteria because these symptoms are not core symptoms specific to PTSD. In the forthcoming International Classification of Diseases, 11th Revision (ICD-11), dysphoric arousal symptoms are excluded from diagnosis of PTSD to reduce comorbidity (Shalev, Liberzon, & Marmar, 2017). Some researchers found that removing dysphoric arousal symptoms from PTSD diagnosis will not cause a substantial change on the prevalence and comorbidity of PTSD (e.g. Elhai, Grubaugh, Kashdan, & Frueh, 2008), which could support retaining dysphoric arousal symptoms in the PTSD diagnosis. Also, some studies found dysphoria arousal symptoms were more highly correlated than PTSD-specific symptoms with general distress (Marshall, Schell, Miles, Corporation, & Monica, 2011). Considering the relationship between dysphoric arousal symptoms and physical functional impairments, our current study provides further support for retaining these symptoms in the PTSD diagnostic criteria.

It is noteworthy that our study is based on a Chinese sample. Psychological factors are implicated in physical illnesses in Chinese traditional medicine. Therefore, many Chinese tend to attribute their psychological distress to physical origins, rather than openly express them (Wang et al., 2000). They usually express their psychological distress by somatization symptoms. This may explain the lack of association between PTSD symptoms and psychosocial HRQoL in our results.

Theoretical and clinical implications of this study should be highlighted. First, it improved our understanding of the clinical heterogeneity of PTSD. The influence of different symptom dimensions of PTSD on HRQoL might occur through different psychopathology processes. To gain a thorough understanding of PTSD and PTSD-related outcomes such as quality of life, studies based on symptom structure models of PTSD might be superior to those based on diagnosis or total symptom scores because they take symptom heterogeneity and more precise measurement of the disorder into consideration. Our results also provide suggestions for clinical practice. According to clinical investigations, treatment for PTSD will lead to improvements in HRQoL (Aversa et al., 2013; Schnurr et al., 2006). Our results suggest that interventions targeting dysphoric arousal symptoms should be developed to improve quality of life among PTSD patients. Specifically, medical or behavioural treatments to improve sleep quality and improve concentration will benefit those troubled by physical functioning impairments following PTSD.

This study has several important limitations. First, both PTSD symptoms and HRQoL were measured by self-report instruments. These results thus need to be replicated by studies using clinician-administered instruments leading to potential same-source bias. Second, our study used a cross-sectional design. Longitudinal studies would further help to clarify the relationships between PTSD symptoms and HRQoL. Finally, the results of our study need to be replicated by studies using populations exposed to a range of trauma types from other cultural backgrounds, since these variables could moderate the results.

Despite the aforementioned limitations, our study provides knowledge on how different symptom dimensions of DSM-5 PTSD are associated with physical and psychosocial HRQoL. By adopting a well-validated seven-factor hybrid model of PTSD, we revealed the important role of symptom heterogeneity in the relationship between PTSD and HRQoL, and in turn provided suggestions for future studies and clinical practice to understand and treat HRQoL impairments in the traumatized population.
