# Supplementary material for: DSM-5 posttraumatic stress symptom dimensions and health-related quality of life among Chinese earthquake survivors
Source: Eur J Psychotraumatol. 2018 May 3;9(1):1468710. doi: 10.1080/20008198.2018.1468710 (PMC5933284; doi:10.1080/20008198.2018.1468710)
Supplement: Supplementary material [file ZEPT_A_1468710_SM3434.zip › Supplementary Figure.docx]

**Supplementary Figure 1** Model diagram of the physical HRQoL model

**Supplementary Figure 2** Model diagram of the psychosocial HRQoL model
