# Supplementary material for: DSM-5 posttraumatic stress symptom dimensions and health-related quality of life among Chinese earthquake survivors
Source: Eur J Psychotraumatol. 2018 May 3;9(1):1468710. doi: 10.1080/20008198.2018.1468710 (PMC5933284; doi:10.1080/20008198.2018.1468710)
Supplement: Supplementary material [file ZEPT_A_1468710_SM3434.zip › Supplementary Table.docx]

**Supplementary Table 1** Means, standard deviations, and Pearson correlations for all the observed variables.

|  | 1 | 2 | 3 | 4 | 5 | 6 | 7 | 8 | 9 | 10 | 11 | 12 | 13 | 14 | 15 | 16 | 17 | 18 | 19 | 20 | 21 | 22 | 23 | 24 | 25 | 26 |
| --- | --- | --- | --- | --- | --- | --- | --- | --- | --- | --- | --- | --- | --- | --- | --- | --- | --- | --- | --- | --- | --- | --- | --- | --- | --- | --- |
| 1.Intrusive thoughts |  |  |  |  |  |  |  |  |  |  |  |  |  |  |  |  |  |  |  |  |  |  |  |  |  |  |
| 2.Nightmares | 0.61 |  |  |  |  |  |  |  |  |  |  |  |  |  |  |  |  |  |  |  |  |  |  |  |  |  |
| 3.Flashbacks | 0.63 | 0.68 |  |  |  |  |  |  |  |  |  |  |  |  |  |  |  |  |  |  |  |  |  |  |  |  |
| 4.Emotional cue reactivity | 0.60 | 0.57 | 0.61 |  |  |  |  |  |  |  |  |  |  |  |  |  |  |  |  |  |  |  |  |  |  |  |
| 5.Physiological cue reactivity | 0.51 | 0.62 | 0.64 | 0.65 |  |  |  |  |  |  |  |  |  |  |  |  |  |  |  |  |  |  |  |  |  |  |
| 6.Avoidance of thoughts | 0.51 | 0.58 | 0.60 | 0.61 | 0.65 |  |  |  |  |  |  |  |  |  |  |  |  |  |  |  |  |  |  |  |  |  |
| 7.Avoidance of reminders | 0.51 | 0.54 | 0.57 | 0.60 | 0.61 | 0.76 |  |  |  |  |  |  |  |  |  |  |  |  |  |  |  |  |  |  |  |  |
| 8.Trauma-related amnesia | 0.29 | 0.38 | 0.36 | 0.32 | 0.42 | 0.44 | 0.43 |  |  |  |  |  |  |  |  |  |  |  |  |  |  |  |  |  |  |  |
| 9.Negative beliefs | 0.34 | 0.38 | 0.43 | 0.40 | 0.46 | 0.44 | 0.45 | 0.50 |  |  |  |  |  |  |  |  |  |  |  |  |  |  |  |  |  |  |
| 10.Distorted blame | 0.32 | 0.40 | 0.40 | 0.37 | 0.41 | 0.44 | 0.44 | 0.49 | 0.58 |  |  |  |  |  |  |  |  |  |  |  |  |  |  |  |  |  |
| 11.Persistent negative emotional state | 0.40 | 0.45 | 0.45 | 0.43 | 0.50 | 0.49 | 0.50 | 0.49 | 0.65 | 0.68 |  |  |  |  |  |  |  |  |  |  |  |  |  |  |  |  |
| 12.Lack of interest | 0.40 | 0.44 | 0.44 | 0.45 | 0.46 | 0.47 | 0.48 | 0.45 | 0.55 | 0.56 | 0.63 |  |  |  |  |  |  |  |  |  |  |  |  |  |  |  |
| 13.Feeling detached | 0.34 | 0.41 | 0.41 | 0.33 | 0.38 | 0.43 | 0.42 | 0.45 | 0.54 | 0.59 | 0.63 | 0.65 |  |  |  |  |  |  |  |  |  |  |  |  |  |  |
| 14.Inability to experience positive emotions | 0.34 | 0.42 | 0.42 | 0.36 | 0.44 | 0.40 | 0.43 | 0.46 | 0.54 | 0.53 | 0.58 | 0.57 | 0.64 |  |  |  |  |  |  |  |  |  |  |  |  |  |
| 15.Irritable/angry | 0.38 | 0.44 | 0.44 | 0.41 | 0.47 | 0.40 | 0.46 | 0.39 | 0.52 | 0.53 | 0.58 | 0.51 | 0.55 | 0.55 |  |  |  |  |  |  |  |  |  |  |  |  |
| 16.Recklessness | 0.33 | 0.42 | 0.42 | 0.32 | 0.42 | 0.44 | 0.43 | 0.49 | 0.50 | 0.56 | 0.55 | 0.55 | 0.63 | 0.55 | 0.56 |  |  |  |  |  |  |  |  |  |  |  |
| 17.Hypervigilance | 0.44 | 0.49 | 0.49 | 0.44 | 0.50 | 0.45 | 0.49 | 0.39 | 0.54 | 0.47 | 0.57 | 0.57 | 0.57 | 0.58 | 0.61 | 0.58 |  |  |  |  |  |  |  |  |  |  |
| 18.Exaggerated startle | 0.49 | 0.55 | 0.55 | 0.51 | 0.57 | 0.51 | 0.52 | 0.37 | 0.51 | 0.45 | 0.53 | 0.52 | 0.50 | 0.53 | 0.57 | 0.48 | 0.64 |  |  |  |  |  |  |  |  |  |
| 19.Difficulty concentrating | 0.41 | 0.45 | 0.45 | 0.41 | 0.46 | 0.45 | 0.47 | 0.40 | 0.47 | 0.46 | 0.56 | 0.52 | 0.54 | 0.60 | 0.57 | 0.55 | 0.61 | 0.67 |  |  |  |  |  |  |  |  |
| 20.Sleep disturbance | 0.41 | 0.47 | 0.47 | 0.47 | 0.42 | 0.43 | 0.43 | 0.38 | 0.42 | 0.37 | 0.48 | 0.47 | 0.44 | 0.48 | 0.53 | 0.42 | 0.51 | 0.57 | 0.65 |  |  |  |  |  |  |  |
| 21.Physical functioning | -0.04 | -0.14 | -0.14 | -0.06 | -0.15 | -0.15 | -0.15 | -0.22 | -0.23 | -0.24 | -0.22 | -0.18 | -0.19 | -0.20 | -0.20 | -0.25 | -0.22 | -0.19 | -0.24 | -0.18 |  |  |  |  |  |  |
| 22.Role-physical | -0.24 | -0.28 | -0.27 | -0.23 | -0.27 | -0.26 | -0.26 | -0.19 | -0.29 | -0.27 | -0.31 | -0.29 | -0.27 | -0.29 | -0.29 | -0.26 | -0.26 | -0.29 | -0.31 | -0.32 | 0.43 |  |  |  |  |  |
| 23.Bodily pain | -0.26 | -0.29 | -0.31 | -0.29 | -0.31 | -0.30 | -0.28 | -0.22 | -0.27 | -0.21 | -0.25 | -0.20 | -0.22 | -0.28 | -0.34 | -0.20 | -0.28 | -0.33 | -0.33 | -0.36 | 0.33 | 0.50 |  |  |  |  |
| 24.General health | -0.22 | -0.24 | -0.25 | -0.21 | -0.23 | -0.22 | -0.20 | -0.19 | -0.23 | -0.16 | -0.19 | -0.25 | -0.21 | -0.23 | -0.28 | -0.19 | -0.27 | -0.30 | -0.31 | -0.36 | 0.36 | 0.47 | 0.53 |  |  |  |
| 25.Role-emotional | -0.19 | -0.25 | -0.24 | -0.22 | -0.23 | -0.23 | -0.23 | -0.25 | -0.28 | -0.27 | -0.31 | -0.29 | -0.27 | -0.30 | -0.30 | -0.23 | -0.27 | -0.30 | -0.34 | -0.36 | 0.40 | 0.73 | 0.44 | 0.44 |  |  |
| 26.Social functioning | -0.24 | -0.30 | -0.29 | -0.25 | -0.30 | -0.32 | -0.29 | -0.28 | -0.33 | -0.31 | -0.35 | -0.31 | -0.34 | -0.34 | -0.38 | -0.35 | -0.35 | -0.33 | -0.34 | -0.33 | 0.44 | 0.49 | 0.56 | 0.48 | 0.49 |  |
| Mean | 1.4 | 1.0 | 1.2 | 1.7 | 1.2 | 1.2 | 1.2 | 0.6 | 0.7 | 0.5 | 0.7 | 0.7 | 0.5 | 0.7 | 0.8 | 0.5 | 0.9 | 1.2 | 1.0 | 1.3 | 79.1 | 64.4 | 76.1 | 60.6 | 45.2 | 79.4 |
| SD | 1.2 | 1.2 | 1.2 | 1.2 | 1.2 | 1.1 | 1.2 | 0.9 | 1.0 | 0.9 | 1.0 | 1.0 | 0.9 | 1.0 | 1.1 | 0.9 | 1.0 | 1.2 | 1.0 | 1.2 | 24.2 | 41.0 | 22.4 | 21.1 | 31.2 | 19.9 |

Note: N=1063.

**Supplementary Table 2** Symptom mappings for confirmatory factor analysis

| PTSD symptoms | Model 1  (*DSM-5*) | Model 2  (*DSM-5*  dysphoria) | Model 3  (*DSM-5*  dysphoric arousal) | Model 4  (Externalizing behaviors) | Model 5  (Anhedonia) | Model 6  (Hybrid) |
| --- | --- | --- | --- | --- | --- | --- |
| B1. Intrusive thoughts | In | In | In | In | In | In |
| B2. Nightmares | In | In | In | In | In | In |
| B3. Flashbacks | In | In | In | In | In | In |
| B4. Emotional cue reactivity | In | In | In | In | In | In |
| B5. Physiological cue reactivity | In | In | In | In | In | In |
| C1. Avoidance of thoughts | Av | Av | Av | Av | Av | Av |
| C2. Avoidance of reminders | Av | Av | Av | Av | Av | Av |
| D1. Trauma-related amnesia | NACM | Dy | NACM | NACM | NA | NA |
| D2. Negative beliefs | NACM | Dy | NACM | NACM | NA | NA |
| D3. Distorted blame | NACM | Dy | NACM | NACM | NA | NA |
| D4.Pervasive negative emotional state | NACM | Dy | NACM | NACM | NA | NA |
| D5. Lack of interest | NACM | Dy | NACM | NACM | An | An |
| D6. Feeling detached | NACM | Dy | NACM | NACM | An | An |
| D7. Inability to experience positive emotions | NACM | Dy | NACM | NACM | An | An |
| E1. Irritability/aggression | Hy | Dy | DA | EB | DA | EB |
| E2. Recklessness | Hy | Dy | DA | EB | DA | EB |
| E3. Hypervigilance | Hy | Hy | AA | AA | AA | AA |
| E4. Exaggerated startle | Hy | Hy | AA | AA | AA | AA |
| E5. Difficulty concentrating | Hy | Dy | DA | DA | DA | DA |
| E6. Sleep disturbance | Hy | Dy | DA | DA | DA | DA |

*Note.* PTSD = Posttraumatic stress disorder. Model 1= the *DSM-5* model; Model 2 = the *DSM-5* dysphoria model; Model 3 = the *DSM-5* dysphoric arousal model; Model 4 = the externalizing behaviors model; Model 5 = the anhedonia model; Model 6 = the seven-factor hybrid model. In = Intrusion; Av = Avoidance; NACM = Negative alterations in cognitions and mood; Hy = Hyperarousal; Dy = Dysphoria; DA = Dysphoric Arousal; AA = Anxious Arousal; EB = Externalizing Behaviors; NA = Negative Affect; An = Anhedonia.

**Supplementary Table 3** Model goodness of fit indices

| Models | χ^2^ | *df* | CFI | TLI | RMSEA | RMSEA 90% CI | BIC |
| --- | --- | --- | --- | --- | --- | --- | --- |
| Model 1 | 981.717 | 164 | .974 | .970 | .068 | .064-.073 | 50167.173 |
| Model 2 | 1131.396 | 164 | .969 | .964 | .074 | .070-.079 | 50318.855 |
| Model 3 | 908.928 | 160 | .976 | .972 | .066 | .062-.071 | 50121.453 |
| Model 4 | 769.916 | 155 | .980 | .976 | .061 | .057-.065 | 50070.179 |
| Model 5 | 789.061 | 155 | .980 | .975 | .062 | .058-.066 | 50046.043 |
| Model 6 | 639.360 | 149 | .984 | .980 | .056 | .051-.060 | 50001.259 |

*Note.* *N* = 1063. CFI = Comparative fit index; TLI = Tucker-Lewis index; SRMR = Standardized root mean square residual; RMSEA = Root mean square error of approximation; CI = Confidence interval; BIC = Bayesian information criterion.

**Supplementary Table 4** Chi - square difference test for comparing nested models

| Models | Δχ^2^ (*df*) | *p* |
| --- | --- | --- |
| Model 1 vs. Model 3 | 64.658(4) | <.001 |
| Model 1 vs. Model 4 | 180.070(9) | <.001 |
| Model 1 vs. Model 5 | 164.988(9) | <.001 |
| Model 1 vs. Model 6 | 285.648(15) | <.001 |
| Model 2 vs. Model 3 | 113.957(4) | <.001 |
| Model 2 vs. Model 4 | 223.621(9) | <.001 |
| Model 2 vs. Model 5 | 215.745(9) | <.001 |
| Model 2 vs. Model 6 | 332.156(15) | <.001 |
| Model 3 vs. Model 4 | 111.174(5) | <.001 |
| Model 3 vs. Model 5 | 98.138(5) | <.001 |
| Model 3 vs. Model 6 | 218.841(11) | <.001 |
| Model 4 vs. Model 6 | 107.099(6) | <.001 |
| Model 5 vs. Model 6 | 122.992(6) | <.001 |

*Note.* *N* = 1063.

**Supplementary Table 5** Standardized factor loadings and factor correlations for the 7-factor PTSD model

| PTSD symptoms | In | Av | NA | An | EB | AA | DA |
| --- | --- | --- | --- | --- | --- | --- | --- |
| B1. Intrusive thoughts | .75 |  |  |  |  |  |  |
| B2. Nightmares | .84 |  |  |  |  |  |  |
| B3. Flashbacks | .85 |  |  |  |  |  |  |
| B4. Emotional cue reactivity | .81 |  |  |  |  |  |  |
| B5. Physiological cue reactivity | .87 |  |  |  |  |  |  |
| C1. Avoidance of thoughts |  | .92 |  |  |  |  |  |
| C2. Avoidance of reminders |  | .91 |  |  |  |  |  |
| D1. Trauma-related amnesia |  |  | .74 |  |  |  |  |
| D2. Negative beliefs |  |  | .83 |  |  |  |  |
| D3. Distorted blame |  |  | .84 |  |  |  |  |
| D4. Persistent negative emotional state |  |  | .92 |  |  |  |  |
| D5. Lack of interest |  |  |  | .85 |  |  |  |
| D6. Feeling detached |  |  |  | .87 |  |  |  |
| D7. Inability to experience positive emotions |  |  |  | .84 |  |  |  |
| E1. Irritable/angry |  |  |  |  | .81 |  |  |
| E2. Recklessness |  |  |  |  | .84 |  |  |
| E3. Hypervigilance |  |  |  |  |  | .85 |  |
| E4. Exaggerated startle |  |  |  |  |  | .85 |  |
| E5. Difficulty concentrating |  |  |  |  |  |  | .89 |
| E6. Sleep disturbance |  |  |  |  |  |  | .81 |
| Av | .87 |  |  |  |  |  |  |
| NA | .72 | .74 |  |  |  |  |  |
| An | .68 | .68 | .92 |  |  |  |  |
| EB | .71 | .70 | .93 | .95 |  |  |  |
| AA | .81 | .74 | .83 | .88 | .94 |  |  |
| DA | .72 | .66 | .76 | .83 | .88 | .93 |  |

*Note. N* = 1063. In = Intrusion; Av = Avoidance; NA = Negative Affect; An = Anhedonia; EB = Externalizing Behaviors; AA = Anxious Arousal; DA = Dysphoric Arousal. All factor loadings and correlations are statistically significant (*p* < .01).
